# Can Fluctuations in Vital Signs Be Used for Pain Assessment in Critically Ill Patients with a Traumatic Brain Injury?

**DOI:** 10.1155/2014/175794

**Published:** 2014-01-22

**Authors:** Caroline Arbour, Manon Choinière, Jane Topolovec-Vranic, Carmen G. Loiselle, Céline Gélinas

**Affiliations:** ^1^McGill University, Ingram School of Nursing, Montreal, Quebec, Canada H3A 2A7; ^2^Centre for Nursing Research and Lady Davis Institute, Jewish General Hospital, Montreal, Quebec, Canada H3T 1E2; ^3^Quebec Nursing Intervention Research Network (RRISIQ), Montreal, Quebec, Canada H3A 2A7; ^4^The Alan Edwards Center for Research on Pain, McGill University, Montreal, Quebec, Canada H3A 0G1; ^5^Department of Anaesthesiology, Centre de Recherche du Centre Hospitalier de l'Université de Montréal (CRCHUM), Université de Montréal, Montreal, Quebec, Canada H2X 0A9; ^6^Trauma & Neurosurgery Program and Keenan Research Center of the Li Ka Shing Knowledge Institute, St. Michael's Hospital, Toronto, Ontario, Canada M5B 1W8

## Abstract

*Background*. Many critically ill patients with a traumatic brain injury (TBI) are unable to communicate. While observation of behaviors is recommended for pain assessment in nonverbal populations, they are undetectable in TBI patients who are under the effects of neuroblocking agents. *Aim*. This study aimed to validate the use of vital signs for pain detection in critically ill TBI patients. *Methods*. Using a repeated measure within subject design, participants (*N* = 45) were observed for 1 minute before (baseline), during, and 15 minutes after two procedures: noninvasive blood pressure: NIBP (nonnociceptive) and turning (nociceptive). At each assessment, vital signs (e.g., systolic, diastolic, mean arterial pressure (MAP), heart rate (HR), respiratory rate (RR), capillary saturation (SpO_2_), end-tidal CO_2_, and intracranial pressure (ICP)) were recorded. *Results*. Significant fluctuations (*P* < 0.05) in diastolic (*F* = 6.087), HR (*F* = 3.566), SpO_2_ (*F* = 5.740), and ICP (*F* = 3.776) were found across assessments, but they were similar during both procedures. In contrast, RR was found to increase exclusively during turning (*t* = 3.933; *P* < 0.001) and was correlated to participants' self-report. *Conclusions*. Findings from this study support previous ones that vital signs are not specific for pain detection. While RR could be a potential pain indicator in critical care, further research is warranted to support its validity in TBI patients with different LOC.

## 1. Introduction

Many patients with a traumatic brain injury (TBI) are unable to self-report their pain in the intensive care unit (ICU) because of altered levels of consciousness (LOC), mechanical ventilation, and/or aphasia [1]. In nonverbal populations, use of behaviors suggestive of pain (a.k.a pain behaviors) such as grimacing, increased muscle tension, protective movements, and noncompliance with the ventilator is recommended for pain assessment [2, 3]. Unfortunately, critically ill TBI patients are commonly under the effects of high doses of sedatives or neuroblocking agents to prevent deleterious elevation of intracranial pressure (ICP). While high doses of sedatives have the potential to attenuate patients' reactivity to sensorial stimuli (including painful ones), neuroblocking agents induce complete paralysis [2]. As such, these drugs make it challenging to use behaviors for pain assessment. Moreover, sedatives (i.e., hypnotic agents and benzodiazepines) and neuroblocking agents have no analgesic properties [4]. For this reason, clinicians cannot rule out the presence of pain in TBI patients receiving them and must rely on signs other than behaviors to perform pain assessment.

Because the autonomic nervous system may be activated during exposure to a painful event, fluctuations in vital signs could be indicative of the presence of pain [5]. In one study by Payen and colleagues [6] with 30 surgical and trauma ICU participants, mean arterial pressure (MAP) and heart rate (HR) were found to increase significantly (*P* < 0.05) during nociceptive procedures (turning and endotracheal (ET) suctioning) compared to nonnociceptive procedures (compression stocking applications and catheter dressing change). However, this study was conducted with unconscious patients only, and the relationship between vital signs' fluctuations and patients' self-report of pain (i.e., the gold standard for pain assessment) could not be examined. Other studies have shown inconsistent findings in relation to vital signs for the detection of pain. In one study with 48 cardiac surgery ICU patients [7], systolic and diastolic blood pressure did not increase significantly during nociceptive procedures (turning and ET suctioning). In another study with 44 surgical, medical, and neurological ICU patients [8], blood pressure and HR increased similarly during a nonnociceptive procedure (eye care) and a nociceptive procedure (turning). In a more recent study [9] with 55 ICU patients with different LOC and various diagnoses (medical, surgical, and trauma with or without TBI), vital signs' fluctuations (i.e., systolic pressure, diastolic pressure, MAP, HR, respiratory rate (RR), capillary saturation (SpO_2_), and end-tidal CO_2_) were not associated with patients' self-report of pain during a nociceptive procedure (turning). Yet, in a similar study [10] with 105 postoperative cardiac ICU patients at different LOC, a moderate positive correlation (*r* = 0.69; *P* ≤ 0.001) was found between HR fluctuations and self-reported pain intensity and a mild negative correlation (*r* = −0.20; *P* ≤ 0.005) was found between SpO_2_ fluctuations and pain intensity during the nociceptive procedure (turning with or without ET suctioning).

Some methodological limitations in the studies mentioned previously are worth noting. First, in all studies, vital signs were documented only once during each assessment period which lasted from one to several minutes. Given that vital signs are recorded every second by ICU monitoring devices to mimic true hemodynamical changes [11], the use of a data collection computer with continuous recording would have enabled more precise calculation of vital signs' fluctuations. Second, most patients in those studies were unable to self-report. The inclusion of patients with different LOC—especially conscious patients able to self-report—would have allowed a better understanding of vital signs' fluctuations in response to pain. Adding to these elements, medical variables such as TBI severity, TBI localization, and those related to the therapeutic regimen (i.e., level of sedation and administration of analgesic and sedative agents) could also affect TBI patients' physiological response to nociceptive exposure, but were not considered in previous studies. Finally, in one study, ICP seemed to increase in six TBI patients exposed to a nociceptive procedure [9], suggesting its validity for pain detection should be further examined.

So far, the validity of relying on vital signs for pain assessment has been examined mainly using two validation strategies called discriminant validation (which consists of comparing fluctuations in vital signs from before to after a nociceptive procedure and a nonnociceptive procedure) and criterion validation (which examines the association between vital signs' fluctuations during a nociceptive procedure and patient's self-report of pain) [12]. Although validity related to the use of vital signs for pain assessment has yet to be supported in empirical studies, it is still recommended by experts [2, 3] as a cue to begin further assessment of pain in patients unable to self-report. As highlighted previously, validation of vital signs for pain detection could be improved through the use of a data collection computer with continuous recording. Given that TBI patients are often uncommunicative in the ICU and that behaviors suggestive of pain may not be discernible in those receiving high doses of sedatives or neuroblocking agents, research is urgently needed to extend and refine the validation of vital signs for the purpose of detecting pain in this specific ICU group.

## 2. Aim and Objectives

This study aimed to validate the use of vital signs for the purpose of pain assessment in critically ill TBI adults. The main study objectives were:

1. for discriminant validation, to describe and compare TBI participants' fluctuations in vital signs (i.e., systolic pressure, diastolic pressure, MAP, HR, RR, SpO_2_, CO_2_, and ICP) recorded across different assessment periods (e.g., 1 minute before, during, and 15 minutes postprocedure), procedures (e.g., nonnociceptive and nociceptive), and LOC (e.g., unconscious, altered, and conscious),
2. for criterion validation, to examine the association between TBI participants' fluctuations in vital signs during the nociceptive procedure and their self-report of pain (in those able to self-report).

A secondary objective of the study was to

(3) explore the potential influence of TBI severity, TBI localization, and therapeutic regimen (e.g., level of sedation and administration of analgesic and sedative agents) on TBI participants' fluctuations in vital signs during the nociceptive procedure.

## 3. Methods

### 3.1. Design, Sample, and Ethics

For this study, a repeated measure within subject design was used to replicate the ICU experience of trauma patients who are commonly submitted to several consecutive procedures. A convenience sample of TBI patients admitted to the ICU of a Level I trauma center in Montreal, Quebec, Canada, was recruited. Patients (when capable of consenting or their legal representative) meeting the following inclusion criteria were considered eligible: (1) 18 years and older and (2) admitted to the ICU following a TBI (with or without other injuries) for more than 24 hours. Patients were excluded if they had (1) a score of 3 (unarousable) on the Glasgow Coma Scale (GCS) [13]; (2) any type of peripheral nerve damage or alteration (e.g., motor paralysis, spinal cord injury, or receiving neuroblocking agents); (3) a documented history of chronic substance abuse in the medical chart; (4) a previous TBI; (5) a diagnosed cognitive deficit or psychiatric condition; and (6) a suspected brain death. Patients were also excluded if they could not be turned in bed. The study was approved by the Ethics Research Committee of the hospital.

### 3.2. Procedure

Sociodemographic characteristics (gender, age, ethnicity, and cause of TBI) and medical information such as severity of injury (injury severity score: ISS) [14], predictor of prognosis (acute physiology and chronic health evaluation: APACHE II score) [15], and LOC (GCS score) [13] were collected for each patient through their medical files. In mechanically ventilated patients, LOC was recomputed with the adapted GCS [16] which takes into account the incapacity of ventilated patients to express themselves verbally in the estimation of the LOC (which is not done in the original GCS). Additional medical information such as TBI severity (i.e., mild, moderate, or severe) and TBI localization (as determined by medical team based on CT scan reading), as well as information related to patient's therapeutic regimen including level of sedation (Richmond agitation sedation scale: RASS) [17] and administration of analgesics and sedatives within four hours prior to data collection (corresponding to the half time of fentanyl and hydromorphone—the two analgesics included in the ICU pain management protocol) were also gathered in the medical files.

TBI participants were observed during two procedures routinely performed in the ICU: (1) noninvasive blood pressure with cuff inflation: NIBP (known as a nonnociceptive procedure) [9] and (2) turning (known as a nociceptive procedure) [18]. For each procedure, participants were observed for 1 minute before (at baseline), during, and 15 minutes postprocedure for a total of six assessment periods. To better understand the influence of LOC on TBI patients' physiologic responses to nociceptive exposure, data collection was repeated in the ICU every time patients changed LOC category based on their GCS score (i.e., unconscious (GCS ≤ 8), altered (GCS between 9–12), or conscious (GCS ≥ 13)) [15]. Specifically, an initial data collection was completed for all participants after recruitment. Then, participants were followed by the research team for any change in LOC categories for up to a month after TBI onset. An additional data collection was performed each time participants changed LOC within this time frame as long as they were still in the ICU, but not if they reverted back to a LOC previously observed.

It is important to mention that both procedures (NIBP and turning) were performed by the ICU nursing staff as this procedure is commonly used in the validation studies of physiologic parameters for the detection of pain in critical care [6–10]. As the duration of the turning procedure was not standardized, the nociceptive procedure (and accordingly the recording of vital signs during the nociceptive procedure) could have lasted more than one minute. Also, ICU nurses were advised to give usual care during data collection and this included giving analgesics and sedatives according to participants' conditions. Although NIBP was taken by ICU nurses, they were instructed to take it on the opposite side of the arterial line to not interfere with the recording of blood pressure (i.e., systolic, diastolic, and MAP). In TBI participants able to communicate, self-reports of pain were collected after each assessment period, but only in those without delirium—as per the Confusion Assessment Method Scale (CAM-ICU) [19] performed prior to data collection.

### 3.3. Variables and Instruments

Vital signs (i.e., systolic pressure, diastolic pressure, MAP, HR, RR, SpO_2_, end-tidal CO_2_, and when available ICP) available through bedside ICU monitoring were recorded continuously using a data collection computer connected to it with a port-serial cable (e.g., Moberg-CNS monitor, PA, USA). All participants wore a five-cable monitoring system (e.g., Dräger monitor software version 5.0 for monitoring of systolic, diastolic, MAP, HR, RR, and ICP) and a finger pulse oximeter (for SpO_2_). In addition, participants wore either a CO_2_ sensor when mechanically ventilated or a CO_2_ nasal cannula when nonmechanically ventilated for recording of end-tidal CO_2_.

Self-reports of pain were obtained by asking conscious participants to report (a) the presence of pain (yes or no) and (b) pain intensity (on a scale of 0–10). Pain intensity was measured using the faces pain thermometer (FPT)—a vertical thermometer ranging from 0 (no pain) to 10 (worst pain imaginable). It includes six faces adapted from the work of Prkachin [20]. The FPT has demonstrated good convergent validation (*r* = 0.80–0.86; *P* ≤ 0.001) with the five-point descriptive pain scale and good discriminant validation (*t* = −5.10; *P* ≤ 0.001) with higher pain intensity score during a nociceptive procedure (i.e., turning) compared to rest in ICU adults [21].

### 3.4. Data Analysis

Descriptive statistics (frequencies, means with standard deviations for normally distributed data, and medians with minimum-maximum values for nonnormally distributed data) were performed for all study variables. To examine the first research objective, means and standard deviations were computed for each vital sign (i.e., systolic pressure, diastolic pressure, MAP, HR, RR, SpO_2_, end-tidal CO_2_, and ICP) recorded during initial data collection at different assessment periods (i.e., 1 minute before, during, and 15 minutes after) and for both procedures (i.e., NIBP and turning). Two-way repeated measures analysis of variance (RM ANOVA) were performed to examine the main effects and the interaction effect of assessment periods and procedures on mean fluctuations in vital signs. According to RM ANOVA assumptions [22], Mauchly's test of sphericity was computed for each vital sign. When Mauchly's test was significant, the greenhouse-Geisser correction was used as it is known to be a more powerful test when sphericity is violated [23]. Post hoc analysis using paired *t*-tests with Bonferroni correction was applied when appropriate [24]. According to LOC, mean fluctuations in vital signs recorded during turning in participants with different LOC at initial and second data collections were computed. Then, paired *t*-tests were performed to compare vital signs' fluctuations observed from baseline to turning at initial data collection in unconscious, altered LOC and conscious participants. Paired *t*-tests were also performed in the subsample of participants involved in a second data collection, but exclusively in conscious participants as only 4 altered LOC participants were included in this subsample—not providing enough power for paired comparison.

For the second objective, mean fluctuations in vital signs of TBI participants who reported pain during turning were compared to mean fluctuations in vital signs of those who reported no pain. Point-biserial correlation (*r*_pb_: for continuous versus dichotomous variables) and Pearson correlation (*r*_*p*_: for continuous versus continuous variables) were performed to examine the relationship between mean vital signs' fluctuations and participants' self-reports of the presence of pain (yes or no) and pain intensity (0–10) during turning.

For the secondary objective of the study, mixed measures ANOVAs were conducted to explore the influence of TBI severity and TBI localization on mean vital signs' fluctuations recorded during both procedures (NIBP and turning). Then, Spearman correlations (*r*_*s*_: for categorical versus continuous variables) and Pearson correlations (*r*_*p*_) were computed to explore the influence of variables related to participants' therapeutic regimen (e.g., level of sedation, analgesics, and sedatives received) on mean vital signs' fluctuations recorded during turning. To facilitate data analysis, analgesics were converted into equianalgesic doses of morphine (e.g., doses that would offer the equivalent amount of morphine) [25]. Sedatives were treated individually as no conversion chart is available.

## 4. Results

### 4.1. Sociodemographic Characteristics, Medical Variables, and Therapeutic Regimen

A total of *N* = 45 participants were included in the study. According to our research objectives, an initial data collection was completed in all participants and a second data collection was completed in a subsample of *n* = 13 participants who changed LOC category within the first month of ICU stay, for a total of 58 data collections. Participants (*N* = 45) involved in the initial data collection were mostly men (*n* = 30, 66.7%), with a mean age of 55.18 years old (SD = 22.08), and were mainly hospitalized for moderate to severe TBI. Participants involved in a second data collection (*n* = 13) were also mostly men (*n* = 8, 61.5%), but had a mean age of 43.00 years old (SD = 19.82), and were all hospitalized for severe TBI. During both initial and second data collections, participants had severe injuries but low risk of intrahospital complications according to a median ISS score of 9 and a median APACHE score below 20 in both samples. Regarding therapeutic regimen, 75% of unconscious participants (*n* = 8) were receiving a combination of fentanyl and diprivan infusions prior to the initial data collection, whereas less than 50% of altered LOC participants (*n* = 21) were receiving fentanyl and/or diprivan infusions and 18.8% of conscious participants (*n* = 16) received subcutaneous (s/c) boluses of hydromorphone. Prior to the second data collection, only 25% of altered LOC participants (*n* = 4) were receiving fentanyl and/or diprivan infusions, while 22.2% of conscious participants (*n* = 9) were receiving s/c boluses of hydromorphone and/or diprivan infusions. Although this was not an exclusion criterion, it is worth noting that participants were not under any type of vasopressors or inotropic drugs and were pacemaker-free at the time of data collection(s). Information about TBI participants' sociodemographic characteristics, medical variables, and therapeutic regimen are available in Tables 1 and 2.

### 4.2. Discriminant Validation of Vital Signs' Fluctuations across Assessments Periods, Procedures, and LOC

During initial data collection, significant fluctuations in diastolic pressure (*F* = 6.087; *P* ≤ 0.01), HR (*F* = 3.566; *P* ≤ 0.05), RR (*F* = 6.228; *P* ≤ 0.01), SpO_2_ (*F* = 5.740; *P* ≤ 0.05), and ICP (*F* = 3.776; *P* ≤ 0.05) were observed across assessment periods (i.e., 1 minute before, during, and 15 minutes postprocedure) (Table 3). Among all vital signs examined, only a significant fluctuation in RR (*F* = 3.872; *P* ≤ 0.05) was found between both procedures (i.e., NIBP and turning). Accordingly, a significant interaction effect between assessment periods and procedures on mean RR fluctuations (*F* = 8.025; *P* ≤ 0.001) was found. Interestingly, a significant interaction effect of assessments and procedures on mean ICP fluctuations (*F* = 6.092; *P* ≤ 0.05) was also observed. Post hoc analysis using paired *t*-tests with Bonferroni correction showed that a significant increase in mean RR values occurred between baseline (i.e., 1 minute before) and turning procedure (*t* = −3.933; *P* ≤ 0.001) and that a significant decrease in mean RR values occurred between turning and 15 minutes postprocedure (*t* = 3.365) (Table 4). In addition, a significant increase in diastolic pressure was also found (*t* = −3.383; *P* ≤ 0.01), but for NIBP only.

According to LOC, no significant fluctuations in vital signs were found between baseline and turning in unconscious TBI participants (*n* = 8) at initial data collection, except for ICP which was found to increase significantly during turning (mean = 5.05; SD = 4.04; *t* = −2.783; *P* ≤ 0.05) (Table 5). Significant increases in RR (Mean = 2.52; SD = 4.18; *t* = −2.558; *P* ≤ 0.05) and HR (mean = 6.27; SD = 5.95; *t* = −3.815; *P* ≤ 0.01) were also observed during turning of altered LOC participants (*n* = 21) at initial data collection. Interestingly, while a significant increase in RR (mean = 9.89; SD = 9.03; *t* = −3.466; *P* ≤ 0.01) was observed during turning of conscious participants (*n* = 16) at initial data collection, no significant changes in vital signs were observed during turning of conscious participants (*n* = 9) at second data collection.

### 4.3. Criterion Validation of Vital Signs with Self-Report of Pain during the Nociceptive Procedure

Thirteen conscious participants were able to report presence of pain (yes or no) and 12 of them were also able to report pain intensity (on the 0–10 FPT) during initial or second data collection. During turning, mean increases in diastolic pressure, MAP, HR, and RR were found in participants who reported pain, while systolic pressure, SpO_2_, and CO_2_ values remained quite stable in those participants (Table 6). However, similar increases in diastolic pressure and MAP were also obtained in participants who reported no pain during turning. Fluctuations in HR (*r*_pb_ = 0.679; *P* ≤ 0.05) and RR (*r*_pb_ = 0.736; *P* ≤ 0.05) were strongly correlated (i.e., Point-biserial correlations) with TBI participants self-report of the presence of pain, but no correlation (i.e., Pearson correlation) was found with pain intensity.

### 4.4. Relationship between Variables Related to TBI Injury or Therapeutic Regimen and Fluctuations in Vital Signs during Turning at Initial Data Collection

Potential influence of TBI severity and TBI localisation on mean fluctuations in vital signs across assessment periods (1 minute before, during, and 15 minutes after) and procedures (NIBP and turning) in participants at initial data collection were explored using mixed measure ANOVAs. No significant differences in vital signs' fluctuations were found according to these variables.

Correlations (i.e., Spearman and Pearson) were performed to explore the relationship between level of sedation and administration of analgesics/sedatives on mean fluctuations in vital signs during turning of participants at initial data collection. No significant correlations between vital signs' fluctuations during turning and TBI participants' level of sedation and administration of sedatives were found. However, a negative significant correlation between equianalgesic doses of morphine received within 4 hours prior to data collection and RR fluctuations was found (*r*_*p*_ = −0.634; *P* ≤ 0.05).

## 5. Discussion

It is widely assumed that fluctuations in vital signs can be indicative of pain [26]. So far, inconsistent findings in previous research do not support the validity to use vital signs for pain assessment in critically ill adults. Yet, they are one of the few observational indicators left for pain assessment in nonverbal TBI patients who are under the effects of high doses of sedatives or neuroblocking agents and who cannot respond behaviorally to pain. This study aimed to further validate the use of vital signs for the purpose of pain assessment in critically ill TBI adults.

Overall, diastolic pressure, HR, RR, SpO_2_, and ICP were found to fluctuate significantly across assessment periods during initial data collection. However, post hoc analysis showed that diastolic pressure fluctuated significantly during the nonnociceptive procedure only, whereas SpO_2_ and ICP fluctuated similarly during both procedures. A possible explanation for this is that vital signs may have been altered by the procedures themselves and not exclusively by the presence of pain. Indeed, turning in bed may cause activation of blood circulation and accordingly influence physiologic parameters such as diastolic pressure, SpO_2_, and ICP which are affected by blood flow [27]. Another possible explanation is that, unlike other ICU patient groups, unconscious and altered LOC TBI patients are exposed hourly to neurological assessments which involve the application of nociceptive stimuli such as nail bed compression [1]. For this reason, and although the nonnociceptive nature of NIBP was previously examined [9], NIBP may still have been perceived by TBI patients as stressful and potentially nociceptive leading to an activation of the autonomic nervous system and causing alteration in vascular resistance and tissue perfusion. This could explain why Young and colleagues [8] found a similar increase in blood pressure and HR during turning (a nociceptive procedure) and eye care (a nonnociceptive procedure that can be stressful especially when performed by someone else) in a sample of neurological ICU patients. Those results combined show that vital signs' fluctuations can be triggered by any type of stimulation in critical care—nociceptive or not.

RR was the only parameter that fluctuated significantly and exclusively during turning at initial data collection. RR was also found to be associated with TBI patients' self-report of pain. This result is consistent with previous findings [28] in which RR was found to significantly increase in a large sample of critically ill soldiers with traumatic injuries (*n* = 2646) reporting the presence of pain. However, fluctuations in RR during a nociceptive procedure could be dependent on patients' LOC. In our study, RR was mostly elevated in conscious and altered LOC TBI patients and remained quite stable in unconscious ones. This result contrasts with previous findings [9, 10] where RR was found to increase significantly during a nociceptive procedure in both conscious and unconscious ICU patients (mostly with medical or surgical diagnoses). Considering that RR does not seem to fluctuate much in unconscious and altered TBI patients and that the significant increase in RR was not replicated within the subsample of conscious participants involved in a second data collection, the validity of RR for pain detection in brain-injured patients needs to be further examined. Interestingly, HR was also found to be correlated with TBI patients' self-report of pain. However, HR was found to fluctuate similarly during the nonnociceptive and the nociceptive procedures. As such, HR could have little utility for the detection of pain in patients unable to self-report.

With respect to therapeutic regimen, a negative correlation between equianalgesic doses of morphine received prior to the nociceptive procedure and mean increases in RR during turning was noted in TBI participants at initial data collection. Considering that TBI patients receiving higher doses of analgesics could be in less pain during a nociceptive procedure, this result further supports the potential usefulness of changes in RR for the detection of pain in TBI patients. Yet, another explanation would be that analgesics (mainly opioids) have a depressive effect on respiratory rate [25]—suggesting once again that vital signs' fluctuations can be influenced by many factors other than pain.

This study was not without limitations. First, the administration of analgesics and sedatives could not be standardized. Secondly and as mentioned previously, patients' vital signs may have been altered by the procedures themselves independently of their type—nociceptive or not. Third, in a complex context of care like the ICU, it is difficult to associate vital signs' fluctuations solely to pain. Indeed, vital signs may also be affected by several emotional states including anxiety and distress. Unfortunately, emotions may be difficult to evaluate in critically ill TBI patients as they often have short attention span and limited cognitive capacities [29].

## 6. Conclusions and Future Directions

Our study findings support discriminant and criterion validation of the use of RR for pain assessment in conscious TBI patients. However, further research is needed to further explore the criterion validation of RR with TBI patients' self-reports of pain (which was possible with only 13 TBI participants in our study). Until then, ICU nurses should use RR exclusively as a cue to begin further assessment of pain until more empirical data are available [2, 3]. Finally, considering the lack of specificity of vital signs for pain detection and because physiological indicators remain relevant for many nonverbal TBI patients in whom behaviors cannot be observed, further efforts at identifying innovative, objective methods of assessing pain in this vulnerable population are needed.

## Figures and Tables

**Table 1:** Sociodemographic characteristics and medical variables of study participants involved in initial data collection (*N* = 45) and subsample involved in a second data collection (*n* = 13)

Variables	Participants involved in initial data collection (*N* = 45)	Subsample involved in a second data collection (*n* = 13)
Age median (min–max)	58 (17–87)	39 (18–77)

Gender *n* (%)		
Male	30 (66.7%)	8 (61.5%)
Female	15 (33.3%)	5 (38.5%)

Ethnicity *n* (%)		
Caucasian	37 (82.2%)	10 (76.9%)
Black	2 (4.4%)	1 (7.7%)
Hispanic	2 (4.4%)	—
First nation	2 (4.4%)	2 (14.4%)
Other	2 (4.4%)	—

Cause of TBI *n* (%)		
Fall	27 (60.0%)	4 (30.8%)
Motor vehicle (MV) accident	9 (20.0%)	5 (38.5%)
Struck by MV	5 (11.1%)	2 (15.4%)
Assault	2 (4.4%)	1 (7.7%)
Other	2 (4.4%)	1 (7.7%)

TBI severity *n* (%)		
Mild	6 (13.3%)	—
Moderate	15 (33.3%)	—
Severe	24 (53.3%)	13 (100.0%)

TBI localisation area *n* (%)		
Frontal	12 (26.7%)	3 (23.08%)
Temporal	12 (26.7%)	1 (7.69%)
Frontotemporal	10 (22.2%)	6 (46.15%)
Temporoparietal	8 (17.7%)	2 (15.38%)
Occipital	3 (6.7%)	1 (7.69%)

LOC category *n* (%)		
Unconscious	8 (17.8%)	—
Altered LOC	21 (46.7%)	4 (30.8%)
Conscious	16 (35.6%)	9 (69.2%)

APACHE II score median (min–max)	14 (6–25)	17 (9–25)

ISS score median (min–max)	9 (9–34)	9 (9–25)

RASS score median (min–max)	−3 (−4 to 1)	−4 (−4 to −2)

APACHE II indicates acute physiology and chronic health evaluation.

ISS indicates injury severity score.

RASS indicates Richmond agitation sedation scale.

**Table 2:** Analgesics, equianalgesic doses of morphine and sedatives administered within 4 hours prior to initial and second data collections^Ψ^ in participants at various LOC.

	Participants involved in initial data collection (*N* = 45)			Subsample involved in a second data collection (*n* = 13)	
	Unconscious GCS ≤ 8 (*n* = 8)	Altered GCS from 9–12 (*n* = 21)	Conscious GCS ≥ 13 (*n* = 16)	Altered GCS from 9–12 (*n* = 4)	Conscious GCS ≥ 13 (*n* = 9)
*Analgesics *					
Fentanyl infusion or I/V bolus (g/h)					
Administration (%)	6 (75.0%)	8 (38.1%)	—	1 (25.0%)	1 (11.1%)
Median dose (min–max)	137.50 (50.00–200.00)	100.00 (50.00–200.00)	—	125.00 (—)	25.00 (—)
Hydromorphone s/c bolus (mg)					
Administration (%)	—	3 (14.3%)	3 (18.8%)	—	2 (22.2%)
Median dose (min–max)	—	1.00 (0.50–2.00)	1.00 (1.00–3.00)	—	3.00 (—)

*Total equianalgesic doses of morphine administered 4 h prior to data collection (mg) *					
Median (min–max)	50.00 (20.00–80.00)	35.00 (2.50–80.00)	5.00 (5.00–15.00)	28.00 (—)	15.00 (10.00–15.00)

*Sedatives *					
Diprivan infusion (mg/h)					
Administration (%)	6 (75.0%)	10 (47.6%)	—	1 (25.0%)	2 (22.2%)
Median dose (min–max)	241.50 (142.00–337.00)	162.00 (31.00–399.00)	—	104.00 (—)	321.00 (192.00–450.00)
Midazolam infusion (mg/h)					
Administration (%)	2 (25.0%)	3 (14.3%)	—	—	—
Median dose (min–max)	6.50 (3.00–10.00)	4.00 (3.500–5.00)	—	—	—
Diazepam I/V bolus (mg)					
Administration (%)	—	2 (9.5%)	—	—	—
Median dose (min–max)	—	15.00 (10.00–20.00)	—	—	—

^Ψ^Of note, initial data collections were performed in all participants (*N* = 45) as they were unconscious (*n* = 8), in altered LOC (*n* = 21), or conscious (*n* = 16). A second data collection was performed in a subsample of participants (*n* = 13) as they were in altered LOC (*n* = 4) or conscious (*n* = 9) bringing the total number of data collections to *n* = 58, of which *n* = 8 were performed in unconscious, *n* = 25 in altered, and *n* = 25 in conscious participants.

**Table 3:** Main effects and interaction effects of assessment periods and procedures on participants' (*N* = 45) mean fluctuations in vital signs at initial data collection.

Conditions	Mauchly's test for sphericity	Two-way RM ANOVA	
	*W*	*F* (Sphericity assumed)	*F* (Greenhouse-Geisser)
Assessment periods—3 levels (before, during, and 15 min after)			
Systolic	0.889	0.241	—
Diastolic	0.867	6.087**	—
MAP	0.808*	—	2.977
HR	0.917	3.566*	—
RR	0.896	6.228**	—
SpO_2_	0.286*	—	5.740*
End-tidal CO_2_	0.959	1.127	—
ICP	0.836	3.776*	—

Procedures—2 levels (NIBP-turning)			
Systolic	NA	0.391	—
Diastolic	NA	1.062	—
MAP	NA	0.471	—
HR	NA	0.040	—
RR	NA	3.872*	—
SpO_2_	NA	0.853	—
End tidal CO_2_	NA	0.127	—
ICP	NA	3.956	—

Interactions (assessment periods × procedures)			
Systolic	0.849*	—	0.235
Diastolic	0.972	0.735	—
MAP	0.901	0.901	—
HR	0.881	2.091	—
RR	0.941	8.025***	—
SpO_2_	0.270*	—	2.307
End tidal CO_2_	0.840	0.423	—
ICP	0.385*	—	6.092*

*P ≤ 0.05; **P ≤ 0.01; ***P ≤ 0.001.

NA indicates nonapplicable.

**Table 4:** Description and post hoc comparison^Ψ^ of vital signs' mean values across assessment periods and procedures in participants (*N* = 45) at initial data collection.

Vital signs mean (SD)	Assessment periods			Post hoc paired-samples *t* tests with bonferroni correction		
	1 min before (baseline)	During	15 min postprocedure	Before→during	During→postprocedure	Postprocedure→before
For NIBP						
Systolic	142.75 (22.40)	143.33 (22.47)	141.94 (23.83)	—	—	—
Diastolic	72.29 (11.83)	76.52 (10.80)	72.64 (13.79)	−3.383**	2.107	1.277
MAP	95.84 (13.45)	98.11 (13.83)	97.06 (15.20)	—	—	—
HR	89.51 (18.92)	89.73 (19.51)	90.36 (19.34)	0.202	0.071	−0.258
RR	21.93 (5.93)	21.61 (7.00)	21.50 (5.46)	0.329	−1.337	0.481
SpO_2_	97.97 (2.15)	96.07 (6.76)	97.97 (2.05)	2.032	−1.961	0.166
End-tidal CO_2_	35.58 (5.95)	35.62 (6.96)	34.67 (6.57)	—	—	—
ICP^ΨΨ^	14.20 (5.28)	13.71 (5.59)	13.97 (5.61)	1.281	−1.271	−0.500

For turning						
Systolic	143.30 (18.85)	143.53 (23.76)	145.23 (25.24)	—	—	—
Diastolic	73.24 (11.90)	76.44 (14.71)	73.41 (14.21)	−1.925	1.967	0.123
MAP	98.16 (12.99)	100.74 (16.33)	97.73 (16.39)	—	—	—
HR	89.08 (17.81)	91.40 (16.33)	90.07 (17.72)	−2.132	1.670	0.693
RR	20.14 (5.31)	24.73 (7.14)	21.67 (5.57)	−3.933***	3.365**	1.547
SpO_2_	97.83 (2.13)	97.37 (2.03)	97.67 (2.36)	2.032	−0.928	−0.552
End-tidal CO_2_	34.86 (6.52)	34.85 (7.96)	34.56 (5.58)	—	—	—
ICP^ΨΨ^	15.04 (5.71)	18.78 (9.83)	13.90 (5.88)	−2.063	2.599	−1.167

^Ψ^Adjusted Bonferroni *P* values: *P ≤ 0.017; **P ≤ 0.003; ***P ≤ 0.000.

^ΨΨ^ICP was available in 16 participants during initial data collection.

**Table 5:** Description and comparison of mean fluctuations in vital signs recorded during turning of participants with different LOC at initial and second data collections.

Vital signs	Participants involved in initial data collection (*N* = 45)						Subsample involved in a second data collection (*n* = 13)			
	Unconscious GCS ≤ 8 (*n* = 8)		Altered GCS from 9 to 12 (*n* = 21)		Conscious GCS ≥ 13 (*n* = 16)		Altered GCS from 9 to 12 (*n* = 4)		Conscious GCS ≥ 13 (*n* = 9)	
	Mean (SD)	*t*	Mean (SD)	*t*	Mean (SD)	*t*	Mean (SD)	*t*	Mean (SD)	*t*
Systolic	−2.74 (18.11)	0.370	2.61 (16.04)	−0.747	−1.93 (12.37)	0.603	1.74 (14.90)	NA	4.22 (10.97)	−1.089
Diastolic	3.30 (8.45)	−0.874	2.45 (9.92)	−1.134	4.21 (12.68)	−1.286	−0.48 (4.05)	NA	5.14 (10.87)	−1.336
MAP	1.53 (11.25)	−0.332	3.36 (10.57)	−1.454	1.91 (9.73)	−0.762	4.49 (11.80)	NA	6.35 (15.01)	−1.197
HR	1.30 (2.86)	−1.202	1.02 (5.19)	−0.900	6.27 (5.93)	−3.815**	−0.17 (1.18)	NA	1.42 (12.93)	−0.310
RR	1.80 (3.83)	−1.150	2.52 (4.18)	−2.558*	9.89 (9.03)	−3.466**	−0.48 (5.60)	NA	1.77 (7.06)	−0.708
SpO_2_	−1.09 (1.27)	2.275	−0.45 (1.05)	1.959	−0.06 (0.92)	0.229	−0.46 (0.09)	NA	0.41 (1.04)	−1.037
End-tidal CO_2_	0.84 (2.20)	−0.940	1.44 (2.48)	−1.235	−2.37 (5.37)	1.324	7.84 (12.20)	NA	4.60 (2.98)	−3.087
ICP^Ψ^	5.03 (4.04)	−2.783*	3.11 (8.27)	−1.188	—	—	—	—	—	—

*P ≤ 0.05; **P ≤ 0.01; ***P ≤ 0.001.

^Ψ^ICP was available in 16 participants during initial data collection.

**Table 6:** Mean vital signs' fluctuations observed during turning procedure in participants who reported the presence or absence of pain.

	Absence of pain during turning (*n* = 4)	Presence of pain during turning (*n* = 9)
Vital signs mean (SD)		
Systolic	−3.68 (18.20)	1.39 (14.61)
Diastolic	10.64 (13.42)	4.73 (15.97)
MAP	7.38 (16.29)	3.34 (14.23)
HR	−8.37 (12.01)	7.08 (7.04)
RR	−1.92 (2.45)	12.64 (8.04)
SpO_2_	0.19 (1.36)	0.01 (0.46)
End tidal CO_2_	2.07 (0.09)	−0.45 (2.17)
ICP^Ψ^	—	—

^Ψ^ICP was not available in conscious participants able to self-report.
